# Secondary Metabolites Produced during the Germination of *Streptomyces coelicolor*

**DOI:** 10.3389/fmicb.2017.02495

**Published:** 2017-12-13

**Authors:** Matouš Čihák, Zdeněk Kameník, Klára Šmídová, Natalie Bergman, Oldřich Benada, Olga Kofroňová, Kateřina Petříčková, Jan Bobek

**Affiliations:** ^1^First Faculty of Medicine, Institute of Immunology and Microbiology, Charles University, Prague, Czechia; ^2^Institute of Microbiology, The Czech Academy of Sciences, Prague, Czechia; ^3^Chemistry Department, Faculty of Science, J. E. Purkinje University, Ústí nad Labem, Czechia

**Keywords:** spore germination, *Streptomyces*, cell signaling, secondary metabolism, albaflavenone, germicidin, chalcone

## Abstract

Spore awakening is a series of actions that starts with purely physical processes and continues via the launching of gene expression and metabolic activities, eventually achieving a vegetative phase of growth. In spore-forming microorganisms, the germination process is controlled by intra- and inter-species communication. However, in the *Streptomyces* clade, which is capable of developing a plethora of valuable compounds, the chemical signals produced during germination have not been systematically studied before. Our previously published data revealed that several secondary metabolite biosynthetic genes are expressed during germination. Therefore, we focus here on the secondary metabolite production during this developmental stage. Using high-performance liquid chromatography-mass spectrometry, we found that the sesquiterpenoid antibiotic albaflavenone, the polyketide germicidin A, and chalcone are produced during germination of the model streptomycete, *S. coelicolor*. Interestingly, the last two compounds revealed an inhibitory effect on the germination process. The secondary metabolites originating from the early stage of microbial growth may coordinate the development of the producer (*quorum sensing*) and/or play a role in competitive microflora repression (*quorum quenching*) in their nature environments.

## Introduction

A large variety of compounds is produced by various microorganisms by means of specialized biosynthetic pathways. Although the special (or secondary) metabolites (Hopwood, 2007; Baltz, 2008; van Keulen and Dyson, 2014) are not essential for growth and reproduction, they often provide the producing organism with a bioactive role (Keller et al., 2005). Reaching further than a cell itself physically can, the small diffusible molecules may give an advantage to its producer by effectively adapting to extracellular conditions to some degree. They may provide defense (or attack), competition, signaling, or interspecies interactions, depending on the environmental cues, thus increasing the likelihood of survival in an inhospitable environment (Brachmann et al., 2013; Martinez et al., 2017). The bioactivity of the small molecules is mostly achieved by affecting transcription in receiving cells (Camilli and Bassler, 2006).

Secondary metabolism is of special interest in *Streptomyces*, a clade of multicellular bacteria that occupies a high position in the developmental hierarchy of bacteria due to their advanced morphology and physiology. Streptomycetes have evolved a plethora of biosynthetic pathways to produce various secondary metabolites, especially signal molecules (see below), or antibiotics (van Keulen and Dyson, 2014). These compounds provide the organism with a competitive advantage, protection from unfavorable living conditions and/or facilitate interspecies interactions (Maxwell et al., 1989).

The genes for the biosynthesis of streptomycete secondary metabolites are mostly clustered and their expression is highly regulated (Bentley et al., 2002; Tanaka et al., 2013). The model *S. coelicolor* possesses the best annotated genome that encodes biosynthetic pathways for more than 20 secondary metabolites (Bentley et al., 2002). The chemical structure has so far been elucidated in less than 30 percent of the compounds, belonging to the following groups of natural substances: polyketides, pyrones, peptides, siderophores, γ-butyrolactones, butenolides, furans, terpenoids, fatty acids, oligopyrroles, and deoxysugars (van Keulen and Dyson, 2014). The remaining 70 percent are called “cryptic compounds” as they are not produced at standard laboratory conditions (Bentley et al., 2002; Ikeda et al., 2003; Ohnishi et al., 2008; Tanaka et al., 2013; van Keulen and Dyson, 2014). To activate these cryptic pathways, streptomycetes are cultivated under non-standard physical and nutritional conditions or co-cultured with other microorganisms (Wakefield et al., 2017). Genetic manipulations within the genes (Luo et al., 2013) or the transfer of the whole biosynthetic gene cluster into a heterologous producer (Kalan et al., 2013; Tanaka et al., 2013) are also commonly used strategies. The successful activation of the biosynthetic pathways often leads to biosynthesis of previously unknown compounds (Ikeda et al., 2003; Ohnishi et al., 2008; Gomez-Escribano et al., 2012; Tanaka et al., 2013). For example, a polyketide alkaloid, coelimycin P1 (so-called yellow pigment), is produced from the *cpk* cryptic gene cluster in *S. coelicolor* (Gomez-Escribano et al., 2012).

The complicated development of streptomycetes requires highly sophisticated control mechanisms mediated by multiple molecules linked to signaling cascades (Kelemen and Buttner, 1998; Claessen et al., 2006; Gao et al., 2012). A widely studied signaling system is *quorum sensing*, in which stimuli are spread within a population and induce appropriate responses (Phelan et al., 2011). Based on the signal assessment, the organism can adapt to its environment and coordinate further development in response to local population densities (Waters and Bassler, 2005). One of the assumptions made in this work is that cellular signaling is also employed in spore germination (see below). However, the nature of the signaling in this developmental phase remains unclear, as do the chemical characteristics and the possible regulatory effect of the produced substances.

Streptomyces undergo a cellular differentiation that resembles the fungal life cycle (Seipke et al., 2012). Their growth starts with germinating spores that develop into a vegetative mycelium of branching hyphae. Subsequent development of aerial hyphae is considered to be a cell response to nutrient depletion; most of the secondary metabolites are formed at this developmental stage (Sello and Buttner, 2008; Seipke et al., 2012). The aerial hyphae are dissected into chains of uninucleoid spores. Spores are subjected to maturation which ensures their survival in unfavorable conditions and allows them to spread into new niches.

The dormant state of spores is characterized by limited metabolic activity or its complete stagnation (McCormick and Flardh, 2012). Subsequent germination is the spore's transition into a metabolically active vegetative phase. Reactivation of the dormant exospore takes place in an aqueous environment. In addition to energy sources (e.g., trehalose) and various nutrients (Ranade and Vining, 1993), the dormant spores of streptomycetes also contain transcriptome which is a remnant of sporulation and spore maturation (Mikulik et al., 2002). The residual pool of mRNA appears to be necessary for the initial germination phase, serving as a template for the early synthesis of proteins, such as chaperones and hydrolases. Whereas chaperones are indispensable in the re-activation of present proteins upon their release from the trehalose milieu (Bobek et al., 2017), hydrolases reconstitute the thick hydrophobic spore cell wall (Bobek et al., 2004; Haiser et al., 2009).

Further development requires the re-activation of the transcriptional apparatus (Paleckova et al., 2006; Mikulik et al., 2008, 2011) controlled by the activity of a set of sigma factors, whose expression takes place from the very beginning of the process (Bobek et al., 2014; Strakova et al., 2014). Genome-wide expression data revealed that the activity of most metabolic pathways is stabilized after the first DNA replication that occurs between 120 and 150 min of germination of *S. coelicolor* (Bobek et al., 2014). After this period, morphologically observable changes, like the first germ tube emerging from the spore, occur (Kelemen and Buttner, 1998; Claessen et al., 2006; Ohnishi et al., 2008).

In the case of non-activated spores, it was found that about 10–20% of spores do not germinate even under optimal incubation conditions (Yoshida and Kobayashi, 1994). Sole spores of *S. viridochromogenes* have been shown to germinate more slowly than in the dense population (Xu and Vetsigian, 2017). This indicates an existence of germination activator produced into the medium. On the other hand, the extract from the *S. viridochromogenes* supernatant has been shown to inhibit the germination of unactivated spores when added prior to incubation (Hirsch and Ensign, 1976a). The inhibitor present was later isolated (along with other congeners) and described as germicidin A (Petersen et al., 1993; Aoki et al., 2011; Ma et al., 2017). The launch of germination within a spore population is stochastic, as was shown not only in streptomycetes (Xu and Vetsigian, 2017) but also in other spore-forming bacteria (van Vliet, 2015). The probability of germination within a population differs between different streptomycete strains; *S. viridochromogenes* and *S. granaticolor* exhibit fast and robust germination whereas *S. coelicolor* and *S. venezuelae* show more complex behavior with a fraction of germlings that stop growing soon after germination (Mikulik et al., 1977; Bobek et al., 2004; Xu and Vetsigian, 2017). Activity of early released compounds, germination activators and inhibitors, may affect the stochasticity of germination in order to adapt the germination strategy to environmental conditions (Petersen et al., 1993; Aoki et al., 2007, 2011; Ma et al., 2017). Since it is considered to be non-productive (Seipke et al., 2012), the initial developmental phase has hitherto not been given sufficient attention. It is nevertheless apparent from the genome-wide expression analysis of *S. coelicolor*'s germinating spores (germlings) performed by Strakova (Strakova et al., 2013), that 163 genes involved in the biosynthesis of secondary metabolites are transcribed during germination (including those cryptic). It is for this reason that we chose to focus on the biosynthetic activities of the germinating spores of *S. coelicolor* in this article. Secondary metabolites produced in this phase would possibly function as germinative signals in the frame of intercellular communication (Rutherford and Bassler, 2012; Brachmann et al., 2013) or may suppress competing microflora.

## Materials and methods

### Preparation of *Streptomyces* spores

*Streptomyces coelicolor* M145 was cultivated on cellophane discs on solid agar plates (0.4% yeast extract, 1% malt extract, 0.4% glucose, 2.5% bacterial agar, pH 7.2) at 28°C for 14 days. Harvested dormant spores were filtered through cotton wool and used to screen for associated secondary metabolites. Spores mixed with 20% glycerol were stored frozen at −20°C.

### Germination and microbial growth

Spores were washed twice in 10 mL sterile distilled water and resuspended in 50 mL NMMP (Kieser et al., 2000), R3 (Shima et al., 1996), or AM (Bobek et al., 2004) liquid medium to a final spore concentration 10^8^ ml^−1^. Glucose, glycerol, or mannitol was used as a carbon source. For boosting synchronicity of the population, spores were incubated for 10 min at 50°C, followed by 6-h germination at 37°C (Hirsch and Ensign, 1976a; Kieser et al., 2000) before screening for produced secondary metabolites.

To prepare samples from the stationary phase of growth, *S. coelicolor* was further cultivated 48 h at 29°C in the same medium. The grown mycelium or supernatant was then used in the screening for produced secondary metabolites.

### Solid phase extraction of secondary metabolites

Secondary metabolites were taken from the culture supernatants extracted using ethyl acetate (Rajan and Kannabiran, 2014), QuEChERS (Schenck and Hobbs, 2004), or solid phase extraction (Kamenik et al., 2010), which was found to be the most suitable and was carried out as follows. An Oasis HLB 3cc 60 mg cartridge (hydrophilic-lipophilic balanced sorbent, Waters, USA) was conditioned with 3 mL methanol (LC-MS grade, Biosolve, Netherlands), equilibrated with 3 mL water (prepared using Milli-Q water purifier, Millipore, USA) and then 3 mL culture supernatant (pH adjusted to 3 with formic acid, 98–100%, Merck, Germany) was loaded. Subsequently, the cartridge was washed with 3 mL water and absorbed substances were eluted with 1.5 mL methanol. The eluent was evaporated to dryness (Concentrator Plus, 2013 model, Eppendorf), reconstituted in 200 μL 50% methanol and centrifuged at 12,000 × g for 5 min.

### LC-MS analyses

LC-MS analyses were performed on the Acquity UPLC system with 2996 PDA detection system (194 - 600 nm) connected to LCT premier XE time-of-flight mass spectrometer (Waters, USA). Five μL of sample was loaded onto the Acquity UPLC BEH C18 LC column (50 mm × 2.1 mm I.D., particle size 1.7 μm, Waters) kept at 40°C and eluted with a two-component mobile phase, A and B, consisting of 0.1% formic acid and acetonitrile, respectively, at the flow rate of 0.4 mL min^−1^. The analyses were performed under a linear gradient program (min/%B) 0/5; 1.5/5; 15/70; 18/99 followed by a 1.0-min column clean-up (99% B) and 1.5-min equilibration (5% B). The mass spectrometer operated in the positive “W” mode with capillary voltage set at +2,800 V, cone voltage +40 V, desolvation gas temperature, 350°C; ion source block temperature, 120°C; cone gas flow, 50 L h^−1^; desolvation gas flow, 800 L h^−1^; scan time of 0.15 s; inter-scan delay of 0.01 s. The mass accuracy was kept below 6 ppm using lock spray technology with leucine enkephalin as the reference compound (2 ng μL^−1^, 5 μL min^−1^). MS chromatograms were extracted for [M+H]^+^ ions with the tolerance window of 0.03 Da, smoothed with mean smoothing method (window size; 4 scans, number of smooths, 2). The data were processed by MassLynx V4.1 (Waters).

### Bioactivity assays

**Figure 1:**
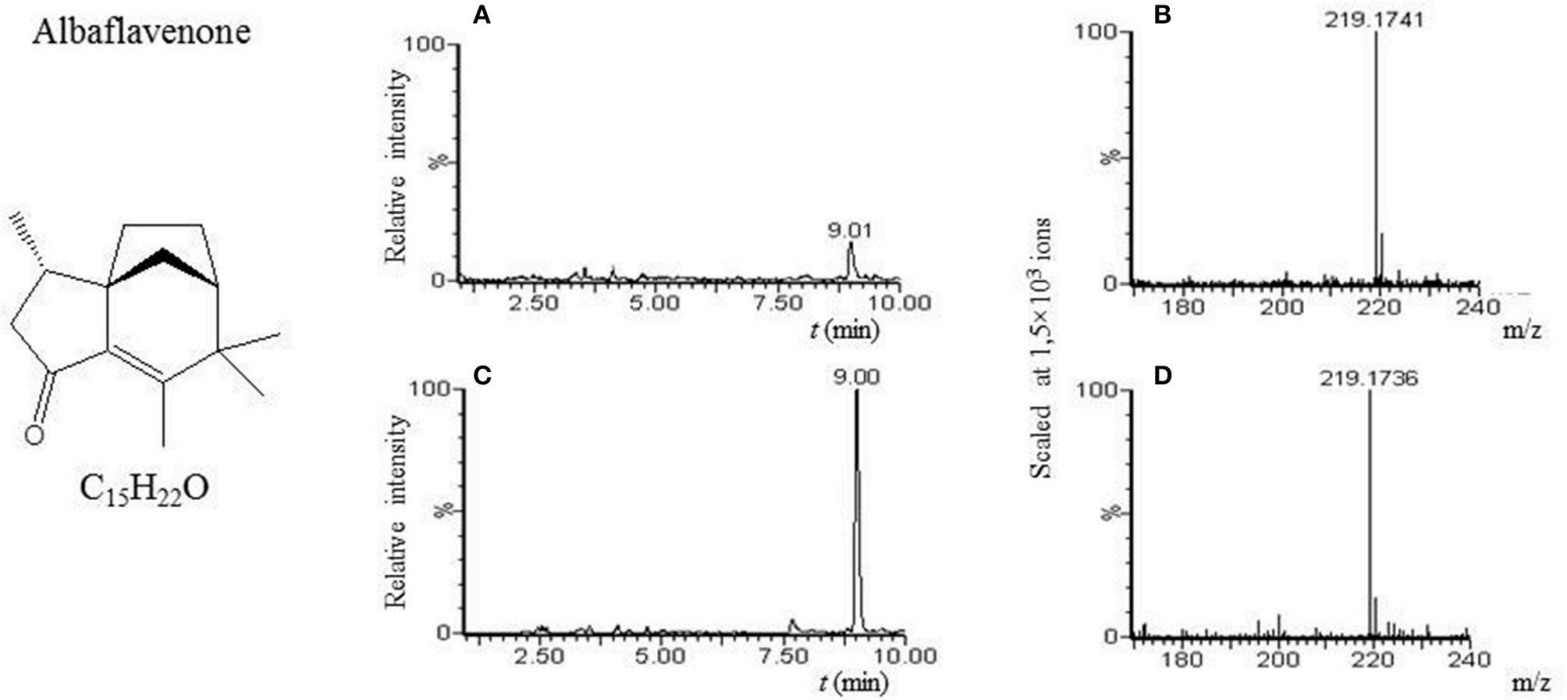
Albaflavenone. **(A)** LC-MS analysis of culture broth extract of *S. coelicolor*'s spores after 6 h germination in AM medium with glycerol. MS chromatogram recorded for *m/z* 219.175. **(B)** Mass spectrum of compound with t_R_ = 9.01 min. Acquired *m/z* 219.1741 corresponds to [M+H]^+^ of albaflavenone with theoretical *m/z* 219.1749. **(C)** LC-MS analysis of hexane extract from supernatant of *S. coelicolor* after 48 h of culture in R3 medium with glycerol (further referred to as albaflavenone-hexane extract). MS chromatogram recorded for *m/z* 219.175. **(D)** Mass spectrum of compound with t_R_ = 9.00 min. Acquired *m/z* 219.1736 corresponds to [M+H]^+^ of albaflavenone ion with the theoretical *m/z* 219.1749.

Although albaflavenone is not commercially available, we have verified its presence using LC-MS in the hexane extract from the supernatant after 48 h of *S. coelicolor'*s growth in R3 medium with glycerol as the carbon source. Cells were centrifuged for 5 min (7,000 × g). The supernatant was extracted three times with 300 mL of *n*-hexane (Lach-Ner, Prague, Czech Republic). The cells were washed in distilled water and mycelial products were extracted with 200 mL of *n*-hexane. Cells were removed by filtration. After separation of the phases, the *n*-hexane layers were pooled (below called as albaflavenone-hexane extract, Figure 1B).

Germicidin A standard was purchased from Cayman Pharma (the Czech Republic), chalcone's standard was obtained from Sigma-Aldrich (Merck, Germany). Dimethylsulfoxid (DMSO) was purchased from Lach-Ner (Czech Republic). On a six-sector culture microtiter plate, ONA medium (1.4% Oxoid nutrient agar, pH 7.2; Kieser et al., 2000) with a linear concentration gradient of germicidin A 0–8 μg mL^−1^ (standard dissolved in sterile distilled water) or chalcone 0–8 μg mL^−1^ (standard dissolved in DMSO), or the albaflavenone-hexane extract (concentration unknown) was poured into three sectors as follows. Pure ONA medium was poured into an inclined plate and allowed to solidify to form a wedge; the plate was then placed horizontally and ONA medium containing selected compound in concentration 8 μg mL^−1^ was poured to form a complementary wedge. The remaining three sectors were filled with either pure ONA medium for control cultivation respective to germicidin A, or ONA medium with DMSO for control cultivation respective to chalcone, or ONA medium with hexane for control cultivation respective to albaflavenone. 10^5^ spores were spread on each sector and incubated 48 h at 29°C. Number of colony-forming units (CFU) was assessed and compared.

### A dehydrogenase activity test

Two milliliter of the albaflavenone-hexane extract was mixed with 5 mL R3 medium with glycerol and used for 6 h germination of 5 × 10^6^ spores. A negative control culture was performed in 5 mL R3 medium with glycerol with 2 mL pure hexane. The dehydrogenase activity test (as described in Burdock et al., 2011) was used to measure metabolic activity of germinating spores by means of triphenyl tetrazolium chloride (TTC). After the germination course, cells were incubated in the presence of TTC and an electron-donating substrate for 1 h. Rising triphenyl formazan (TF) was extracted using ethanol and its concentration was determined colorimetrically by measuring the optical density at wavelength of 484 nm. The absorbances were compared between the tested and negative control samples.

### Scanning electron microscopy

*Streptomyces* spores were fixed with 3% glutaraldehyde overnight at 4°C. The fixed spores were extensively washed and then allowed to sediment at 4°C overnight onto circular coverslips treated with poly-L-lysine. The coverslips with attached spores were dehydrated through an alcohol series followed by absolute acetone and critical point dried from liquid CO_2_ in a K850 Critical Point Dryer (Quorum Technologies Ltd, Ringmer, UK). The dried samples were sputter-coated with 3 nm of platinum in a Q150T Turbo-Pumped Sputter Coater (Quorum Technologies Ltd, Ringmer, UK). The final samples were examined in a FEI Nova NanoSEM scanning electron microscope (FEI, Brno, Czech Republic) at 5 kV using CBS and TLD detectors. Beam deceleration mode of scanning electron microscope was used in some cases for minimization of charging artifacts.

## Results

### *In silico* analysis of the expressed secondary metabolite biosynthetic genes during germination of *S. coelicolor*

Genes that are expressed in the consecutive time points of germination have been reported by Strakova (Strakova et al., 2013). From their dataset we selected genes whose products are involved in the biosynthesis of secondary metabolites by *S. coelicolor*, according to the StrepDB database (http://strepdb.streptomyces.org.uk), where the annotated *S. coelicolor* genes are categorized into metabolic groups. We updated the list of secondary metabolites with regard to newly published findings (Zhao et al., 2009; van Keulen and Dyson, 2014). The resulting list of genes is summarized in Table 1. Predicted secondary metabolites, whose respective genes are expressed during germination, include polyketides, pyrones, peptides, siderophores, terpenoids, oligopyrroles, and fatty acids.

**Table 1:** An overview of biosynthetic genes expressed during germination, according to Strakova et al. (2013).

**Secondary metabolites**	**Biosynthetic (SCO) genes expressed during germination**
Actinorhodin and related congeners	*0331; 3978; 4280; 5072-5079; 5081-5086; 5088-5090*
Albaflavenone	*5223*
Calcium-dependent antibiotic (CDA)	*3210; 3212; 3214; 3220-3221; 3223; 3225; 3227; 3228; 3230-3232; 3234-3237; 3239; 3241-3242; 3246; 3249*
Coelibactin	*7682-7684; 7686-7687; 7689-7691*
Coelichelin	*0490-0498*
Coelimycin P1	*6273-6275; 6277-6287*
Desferrioxamines B, E, G_1_ a D_1_	*2783-2784*
Eicosapentaenoic acid (EPA)	*0124-0125*
Flaviolin, THN	*1206*
Geosmin	*6073*
Hopanoids, ATBH	*6759-6763; 6765; 6767; 6769; 6771*
Isorenieratene, β-carotene	*0187-0191*
Streptorubin B, undecylprodigiosin	*0126-0127; 5877-5878; 5881; 5891-5894; 5898*
Triketid pyrones	*7670-7671*
Tw95a	*5314; 5318; 5320*

### Secondary metabolites produced by *S. coelicolor* during germination and/or in the stationary phase

The fact that genes whose products are involved in the secondary metabolite biosynthesis were transcribed during germination encouraged us to investigate whether germinating spores produce respective compounds up to the 6th hour of their development. For this purpose, we performed an LC-MS analysis of the culture supernatants. In order to ensure that any detected compound is synthesized *de novo* during germination, we included dormant spores (non-activated) as negative control samples in the analysis. As a positive control that tests effectiveness of our detection method, samples from the sporulation phase were included too. The compounds detected only in the sporulation phase (after 48 h of cultivation) but absent from the germination are listed in Table 2.

**Table 2:** Secondary metabolites detected in the stationary phase of growth.

**Secondary metabolite**	**Medium (carbon source)**	**Molecular formula**	**Theoretical mass (*m*/*z*)**	**Acquired mass (*m*/*z*)**	**Mass error (ppm)**
γ- actinorhodin	R3, AM, NMMP (glycerol, mannitol, glucose)	C_32_H_22_O_14_	631.1090	631.1081	1.40
Actinorhodinic acid	R3, AM, NMMP (glycerol, mannitol, glucose)	C_32_H_26_O_16_	667.1300	667.1289	1.60
Albaflavenone	R3 (glycerol)	C_15_H_22_O	219.1736	219.1749	5.90
CDA	R3 (glycerol)	C_67_H_78_N_14_O_26_	1495.5290	1495.5292	0.10
Coelimycin P1	R3 (glycerol)	C_17_H_20_N_2_O_4_S	349.1222	349.1229	2.00
Chalcone	R3 (glycerol/mannitol)	C_15_H_12_O	209.0960	209.0966	2.90
Desferrioxamine B	R3 (glycerol/mannitol) AM (glycerol/mannitol)	C_25_H_48_N_6_O_8_	561.3625	561.3612	2.30
Desferrioxamine D_1_	AM (glucose/mannitol)	C_27_H_50_N_6_O_9_	603.3711	603.3718	1.20
Desferrioxamine E	R3 (glycerol/mannitol) AM (glucose/glycerol) NMMP (glucose/mannitol)	C_27_H_48_N_6_O_9_	601.3561	601.3561	0.00
Desferrioxamine G_1_	AM (glucose/glycerol)	C_27_H_50_N_6_O_10_	619.3677	619.3667	1.60
Germicidin A	R3 (glycerol/mannitol) AM (glucose/mannitol)	C_11_H_16_O_3_	197.1170	197.1178	4.10
Germicidin B	R3 (glycerol/mannitol) NMMP (glucose/mannitol)	C_10_H_14_O_3_	183.1016	183.1021	2.73
Kalafungin	R3 (mannitol)	C_16_H_12_O_6_	301.0709	301.0714	1.00
Streptorubin B	R3 (glycerol/mannitol) AM (glucose/glycerol)	C_25_H_33_N_3_O	392.2696	392.2702	1.50
Undecylprodigiosin	R3 (glycerol/mannitol) AM (glucose/glycerol)	C_25_H_35_N_3_O	394.2866	394.2858	2.00

**Figure 2:**
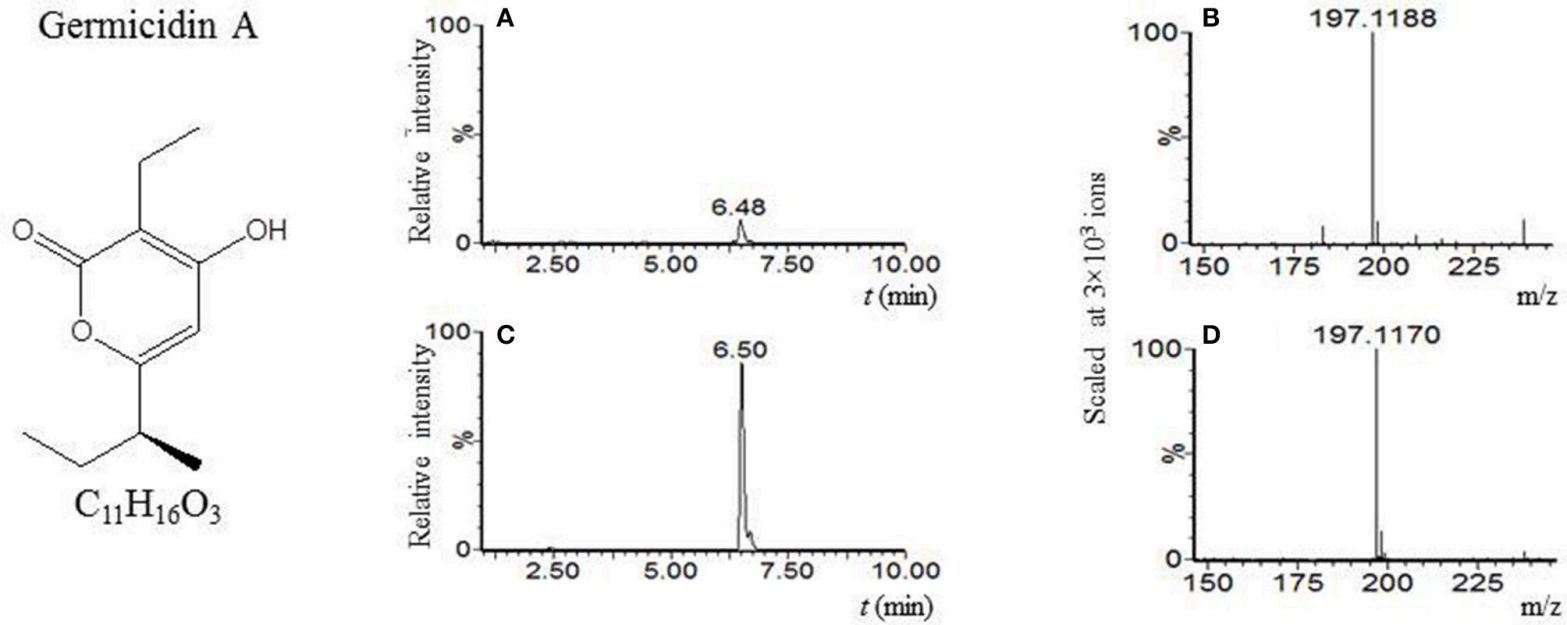
Germicidin A. **(A)** LC-MS analysis culture broth extract of *S. coelicolor*'s spores after 6 h germination in R3 medium with glycerol. MS chromatogram recorded for *m/z* 197.118. **(B)** Mass spectrum of compound with t_R_ = 6.48 min. Acquired *m/z* 197.1188 corresponds to [M + H]^+^ of germicidin A with the theoretical *m/z* 197.1178. **(C)** LC-MS analysis of germicidin A authentic standard. MS chromatogram recorded for *m/z* 197.118. **(D)** Mass spectrum of germicidin A. Acquired *m/z* = 197.1170 corresponds to [M+H]^+^ of germicidin A with the theoretical *m/z* = 197.1170.

**Figure 3:**
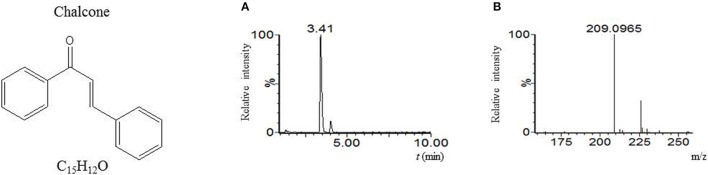
Chalcone. **(A)** LC-MS analysis culture broth extract of *S. coelicolor*'s spores after 6 h germination in R3 medium with glycerol. MS chromatogram recorded for *m/z* 209.097. **(B)** Mass spectrum of compound with t_R_ = 3.41 min. Acquired *m/z* 209.0965 corresponds to [M+H]^+^ of chalcone with the theoretical *m/z* = 209.0966.

Our LC-MS measurements revealed, however, that the *S. coelicolor*'s germlings produce three different compounds with masses corresponding to the sesquiterpenoid antibiotic albaflavenone, the polyketide germicidin A, and chalcone (Table 3; Figures 1–3, respectively). Furthermore, the identity of germicidin A was confirmed by comparing the actual retention time with the original standard obtained from Cayman Pharma (Figure 2).

**Table 3:** Secondary metabolites produced during germination.

**Secondary metabolite**	**Medium (carbon source)**	**Molecular formula**	**Theoretical mass (*m*/*z*)**	**Acquired mass (*m*/*z*)**	**Mass error (ppm)**
Albaflavenone	AM (glycerol)	C_15_H_22_O	219.1741	219.1749	3.70
Germicidin A	R3 (glycerol/mannitol) AM (glucose/mannitol)	C_11_H_16_O_3_	197.1188	197.1178	5.10
Chalcone	R3 (glycerol/mannitol)	C_15_H_12_O	209.0965	209.0966	0.50

#### Actinorhodin production

Actinorhodin compounds (actinorhodinic acid and γ-actinorhodin) were detected in both dormant and germinating spores, as well as in samples from the stationary phase of growth (Table 2). Streptomycetes produced these compounds after 48 h of cultivation in R3, AM, and NMMP medium, regardless of carbon source (glucose, glycerol, or mannitol). Although the suspensions of dormant spores were washed in distilled water, we found that dormant spores also contained these blue pigments. We also found actinorhodinic acid and γ-actinorhodin in the cell-free supernatant after spore germination in AM medium. All supernatants containing the blue pigments also exhibited a pH-dependent color change.

### pH-dependent biosynthetic activity of germlings

**Figure 4:**
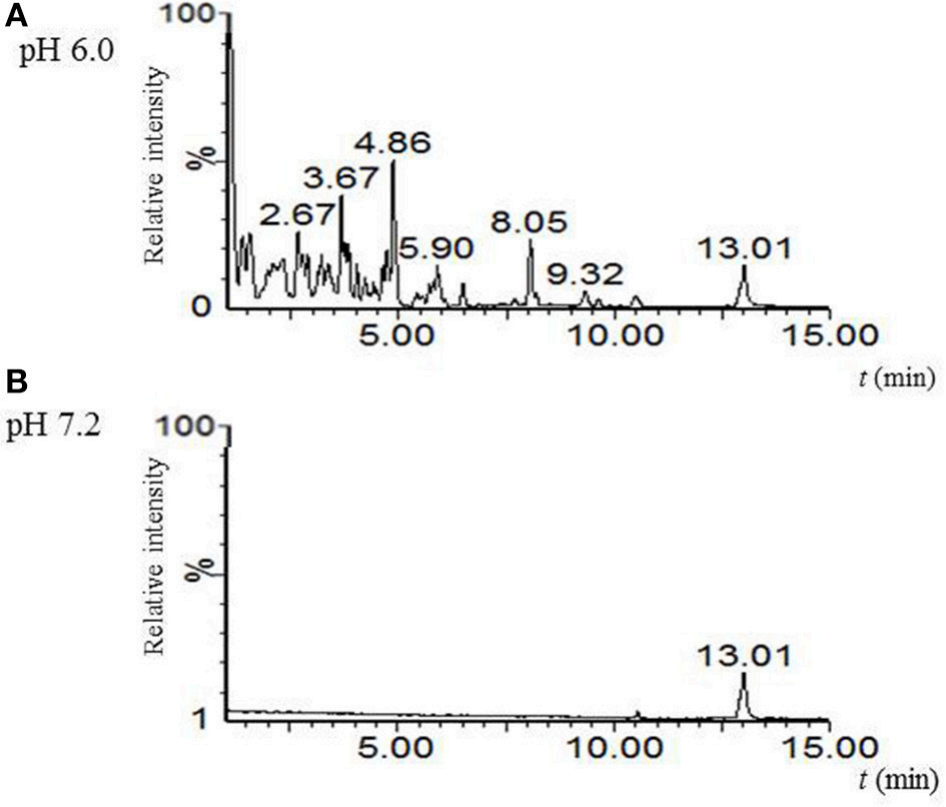
pH-dependent biosynthetic activity of germlings. Comparison of base peak MS chromatograms acquired for culture broth extracts of *S. coelicolor*'s spores after 6 h germination in R3 medium with glycerol differing at pH; **(A)** pH 6.0; **(B)** pH 7.2.

Biosynthesis of albaflavenone involves the activity of cytochrome P450 (CYP170A1) which, depending on pH, can either act as a monooxygenase (pH 7.0-8.2) or as a farnesene synthase (5.5–6.5) (Zhao et al., 2009). Whereas β-farnesene was not synthesized in cultures in R3, NMMP, and AM medium at pH 7.0–7.2, we showed that the germlings produce a substance corresponding to albaflavenone. To see whether β-farnesene is produced in more acidic conditions, we performed a germination experiment in R3 medium with glycerol at pH 6.0. Secondary metabolites were extracted by the solid phase extraction, followed by the LC-MS analysis. To compare, we used an extract after 6 h of cultivation at pH 7.2 without changing the other conditions. Although the direct production of β-farnesene was not found, it is clear from the total ionic and base peak chromatograms that under different pH conditions *S. coelicolor* produces a different spectrum of substances (Figure 4). Further analysis was beyond the scope of this manuscript.

### Biological effects of albaflavenone, germicidin A, and chalcone

#### Albaflavenone

**Figure 5:**
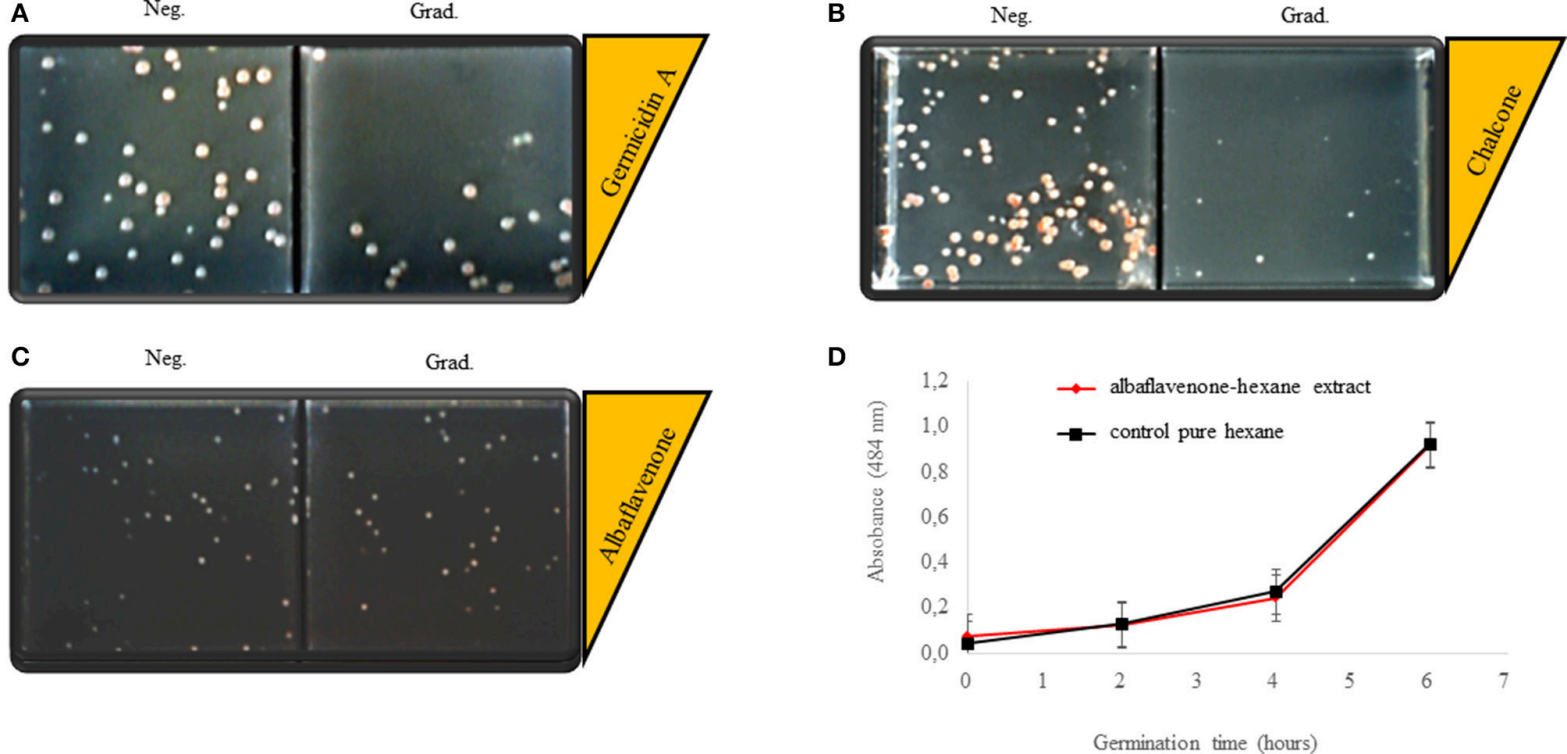
Biological activities of germicidin A, chalcone, and albaflavenone-hexane extract. Bioassays **(A–C)** were performed on the six-sector cultivation titration plates in two sets with negative control triplets (see Methods for more details), representative pictures are shown. **(A)** (Neg.) negative control culture on ONA medium without germicidin A. (Grad.) culture on ONA medium with a linear concentration gradient of germicidin A (0–8 μg mL^−1^ respectively to the yellow wedge). **(B)** (Neg.) negative control culture on ONA medium with a DMSO gradient without chalcone. (Grad.) culture on ONA medium with a linear concentration gradient of chalcone (0–8 μg mL^−1^ respectively to the yellow wedge). **(C)** (Neg.) negative control culture on ONA medium with a pure hexane gradient. (Grad.) culture on ONA medium with a linear concentration gradient of the albaflavenone-hexane extract respective to the yellow wedge. **(D)** A dehydrogenase activity test. Spores germinated in the presence of the albaflavenone-hexane extract (red) or in the presence of pure hexane (black). Metabolic activity was measured at denoted time points by optical density at 484 nm.

Since albaflavenone was not commercially available, its possible effect on germination was tested using the albaflavenone-hexane extract (see Methods). A negative control, which contained pure hexane, was included in the experiment. No quantitative or phenotypic changes were observed in the tested conditions (Figure 5C). To verify this result, a dehydrogenase activity test was performed (Figure 5D). Metabolic activity of living cells that are present in the medium during germination was determined as a function of their dehydrogenase activity, proportional to the concentration of rising TF measured by the optical density at 484 nm (see Methods for details). The optical density increased in time during germination at the same rate in both tested and control samples; the albaflavenone-hexane extract created no observable effect.

The biological effect of the other two secondary metabolites on germination was examined using standards of gemicidin A (Cayman Pharma) and chalcone (Sigma-Aldrich). Experiments were performed on six-section cultivation titration plates (Gama Group). Three fields in one column contained ONA medium with a gradient of tested compound (0–8 μg mL^−1^) and pure medium without the tested substance was poured into the fields in the other column as negative controls. Results were evaluated in terms of the number of colony forming units (CFU) and phenotypic changes.

#### Germicidin A

Germicidin A clearly inhibited the germination of *S. coelicolor* from the concentration of 4 μg mL^−1^ (Figure 5A). The average number of colony-forming units (germinating spores) was 20 on an ONA medium with the linear gradient of germicidin A at 4 μg mL^−1^ and lower; 60 colonies were grown without germicidin A as a negative control. The tested colonies were of the same shape and size as their controls. Actinorhodin production was quantitatively the same.

#### Chalcone

**Figure 6:**
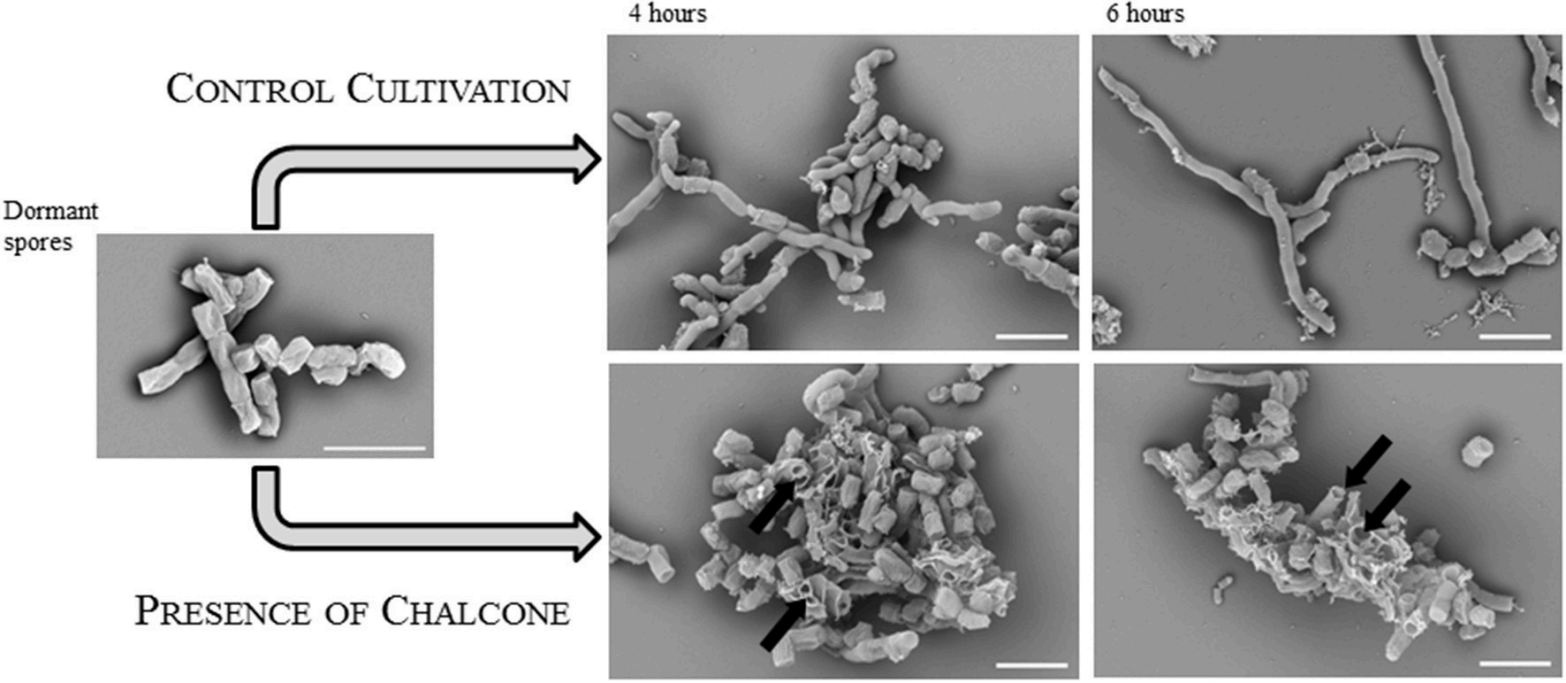
An inhibitory effect of chalcone on spore germination. Electron microscopic images of dormant (left) and germinating spores after two (middle) and four (right) hours of cultivation in the liquid R3 medium. Control cultivation was performed in the absence of chalcone. In the presence of chalcone (in a concentration of 80 μg.mL^−1^), spores or germ tubes are disrupted (indicated by black arrows). The white bars indicate 2.5 μm.

Our initial experiments showed that chalcone suppressed germination of *S. coelicolor* in concentrations down to 8 μg mL^−1^ on the solid medium (Figure 5B). The average number of CFU was 20 on a medium with a chalcone gradient (0–8 μg mL^−1^), contrary to 70 colonies on the chalcone-free medium. The size of the colonies was inversely proportional to the chalcone concentrations; the colonies were significantly smaller compared to the negative control, suggesting a slower germination rate and/or vegetative growth. Moreover, in the presence of chalcone, actinorhodin was not produced throughout the whole cell cycle. The effect of chalcone was additionally examined in R3 liquid medium. Whereas the concentration 8 μg mL^−1^ revealed to be subinhibitory, the chalcone concentration of 80 μg mL^−1^ completely suppressed the development as could be seen in electronmicroscopic images taken from the germination course in the 4th and 6th hour of cultivation Figure 6. As could be seen in the image, the developing germ tubes disrupt in the presence of chalcone, leaving empty cell envelopes.

## Discussion

Although many secondary metabolites of streptomycetes have been discovered, they were most often isolated from the stationary phase of growth, i.e., in the context of the formation of aerial mycelium (Janecek et al., 1997; Kieser et al., 2000; van Keulen and Dyson, 2014). However, our *in silico* search within the gene expression data (Strakova et al., 2013; Bobek et al., 2014) revealed a number of genes (including those cryptic) responsible for the biosynthesis of secondary metabolites to be expressed during the course of *S. coelicolor'*s germination (see Table 1); especially genes responsible for the synthesis of desferrioxamines (sco2783-2784), gray spore pigment (sco5314, sco5318, sco5320), or yellow coelimycin P1 (sco6273-6276, sco6277-6287, so called *cpk* cryptic gene cluster) (Lakey et al., 1983; Gomez-Escribano et al., 2012). We therefore focused our work on whether germinating spores are capable of activating the respective biosynthetic pathways and producing any compound within the 6 h of germination.

Metabolites that could be bound to the spore surface or present in germination medium were identified by means of the LC-MS analysis. Simultaneously, the secondary metabolites produced during the sporulation phase and those associated with the dormant spores were also included in the analysis in order to state whether the compounds found in the samples from germlings were synthesized *de novo*. The cultivations were carried out in three different liquid media (R3, NMMP, and AM, see Methods). The nutritionally rich medium R3 was chosen for the capacity of *S. coelicolor* to produce a number of structurally distinct secondary metabolites in it, such as actinorhodin (Shima et al., 1996) or coelimycin P1 (Gomez-Escribano et al., 2012). In contrast to the R3 medium, the minimal liquid medium NMMP, a poorer medium in which streptomycetes produce fewer secondary metabolites (Hodgson, 1982), was also used. The reason is that NMMP enables the testing of the effects of various ions and nutrients on the production of secondary metabolites. The AM medium containing 20 amino acids was also implemented into our experiments as it had been specifically designed for germination experiments and was used throughout the whole genome expression analyses (Bobek et al., 2004; Strakova et al., 2013). It is known that the presence of different carbon and energy sources in the medium qualitatively affects secondary metabolism (Janecek et al., 1997). That is why the presence of various sugars—glucose, glycerol, and mannitol—in all three media types was tested. In accordance with previously published data (Kieser et al., 2000), both glycerol and mannitol were revealed to be a more suitable source of carbon for secondary metabolites production in the R3 medium in our experiments. Mannitol and glucose exhibited a higher capacity for secondary metabolism when NMMP medium was used and glycerol was shown to have a higher capacity in cases where AM medium was used. Our results also showed that both nutritionally richer media R3 and AM are more suited to germination and the production of secondary metabolites than the minimal NMMP medium, probably due to the presence of Ca^2+^ ions (Eaton and Ensign, 1980; Lakey et al., 1983), L-amino acids (Hirsch and Ensign, 1976b), or various carbon sources (Romero-Rodriguez et al., 2016) in the richer media.

The elution methods with ethylacetate (Rajan and Kannabiran, 2014) or the QuEChERS (Schenck and Hobbs, 2004) were not the most appropriate for isolation of secondary metabolites. Therefore, solid phase extraction (Kamenik et al., 2010) was applied with optimization for streptomycete secondary metabolites. These extracts from supernatants of *S. coelicolor*'s cultures were used for LC-MS analyses.

### Secondary metabolites of *S. coelicolor* not produced during germination

Most of the secondary metabolites, whose biosynthetic genes had previously been shown to be expressed during germination (Strakova et al., 2013), were not detected in samples from germination. These include 21 genes (including sco3230-3232 that encode CDA peptide-synthetase I-III) from the *cda* gene cluster [encoding synthesis of the calcium-dependent-antibiotic (CDA)], the cryptic gene cluster *cpk* (genes sco6273-6288, encoding synthesis of a polyketide antibiotic coelimycin P1), genes sco5314, sco5318, and sco5320 (encoding synthesis of the gray spore pigment), and genes sco5877-5878, sco5881, sco5891-5894, and sco5898 from the so-called *red* gene cluster (encoding synthesis of undecylprodigiosin), and genes sco2783-2784 from the *desABCD* cluster (sco2782-2785, controlling the synthesis of desferrioxamines). Despite the respective gene expression, biosynthesis of more complex secondary metabolites may not occur, since gene expression is only a requirement for biosynthesis and not evidence of it actually taking place.

On the other hand, we found two actinorhodin congeners - actinorhodinic acid and γ-actinorhodin (Bystrykh et al., 1996; Okamoto et al., 2009) in all tested developmental phases, i.e., stationary phase, dormant and germinating spores. In germinating spores, expression of several involved genes: sco5072-5086, sco5088, and sco5090 had been found (Strakova et al., 2013). Despite the detected expression, we cannot exclude the possibility that the presence of actinorhodin originates from the stationary phase rather than from *de novo* synthesis in germination (therefore the compounds are not listed in Table 3). The reason is that actinorhodin (as well as other aromatic pigments derived from the type II and type III polyketide synthases) is known to be bound on the spore envelopes throughout dormancy (Davis and Chater, 1990; Bystrykh et al., 1996; Funa et al., 1999; Tahlan et al., 2007). This was also confirmed by the actinorhodin detection in our samples of dormant spores even after several washings.

### Secondary metabolites of *S. coelicolor* produced during germination

The latest study on the topic (Xu and Vetsigian, 2017) concludes that the germination of *S. coelicolor* M145 may be positively or negatively affected by unknown substances produced by the germlings themselves or by other streptomyces species (e.g., *S. venezuelae*). The results presented here unveiled that *S. coelicolor* produces three secondary metabolites during germination, belonging to the terpenoids (albaflavenone) and polyketides (germicidin A, chalcone). As these compounds have not been detected in dormant spore extracts, we assume that they are produced *de novo* during germination. They show a variety of biological effects and thus perhaps help *S. coelicolor* suppress competitive microflora or coordinate its own development at the early stage of development. The biosynthetic pathways of the detected substances encompass only a few simple reaction steps that do not require complicated precursors and whose biosynthetic genes are expressed during germination, as can be found in gene expression data (Strakova et al., 2013). In contrast, structurally complex metabolites (like the CDA) were not detected in germination (see above).

### Albaflavenone

Tricyclic sesquiterpenoid albaflavenone has an aroma similar to geosmin (Gerber and Lechevalier, 1965; Gurtler et al., 1994). Its biosynthesis requires only two genes: sco5222-5223 (Moody et al., 2012). The expression of these genes can be suppressed by cAMP-receptor protein, Crp, which also occurs in other bacteria (e.g., in *Escherichia coli*). The cAMP-Crp control system is a key regulator of germination, secondary metabolism, and further development of *S. coelicolor* (Derouaux et al., 2004; Gao et al., 2012; Bobek et al., 2017). The system also influences the expression of biosynthetic gene clusters in *S. coelicolor* that extend beyond albaflavenone actinorhodin, prodigiosin, CDA, and coelimycin (Gao et al., 2012).

So far, the production of albaflavone in streptomycetes has been described only in the stationary growth in *S. albidoflavus* (Gurtler et al., 1994), *S. coelicolor, S. viridochromogenes, S. avermitilis, S. griseoflavus, S. Ghanaensis*, and *S. albus* (Moody et al., 2012). However, the expression data analysis shows that the gene sco5223 is activated in germination (Strakova et al., 2013), indicating the possible formation of this metabolite during the initial 6 h of cultivation. We actually found a substance corresponding to albaflavenone in the germination sample in the AM medium with glycerol. The compound was also detected in samples from the stationary phase (positive control in R3 medium with glycerol), but not samples from dormant spores, indicating its germination-associated *de novo* synthesis.

Albaflavenone is not commercially available, which is why we used hexane extracts, where the compound was detected by LC-MS, for testing its biological activities. We did not see any effect, however. On the other hand, it can be assumed that the albaflavenone produced during the germination provides an advantage in a highly competitive soil environment because it has a demonstrable antibacterial effect on *Bacillus subtilis* at the concentration of 8 μg mL^−1^ (Gurtler et al., 1994). Moreover, if albaflavenone was incorporated into the hydrophobic envelope of spores, as other terpenoids do incorporate into the lipophilic membrane layers, it would affect the permeability of the envelopes leading to an intense water influx into spores, thereby accelerating their germination. If we reason that the thickness of the hydrophobic spore envelope is not unified (Lee and Rho, 1993), then the water influx comes into different spores in a different intensity, and therefore, naturally, germination is a non-synchronous process (Hirsch and Ensign, 1976a; Xu and Vetsigian, 2017). The spores already germinated would produce albaflavenone as a signal that environmental conditions are appropriate for the growth of the whole population.

β-farnesene is a sesquiterpene relative to albaflavenone with a wide range of bioactivities (Gibson and Pickett, 1983; Avé et al., 1987) that serves as a precursor of a number of biosynthetic pathways, including the geosmin synthesis. The synthesis of both albaflavenone and β-farnesene is dependent on the type of activity carried out by cytochrome P450 (CYP170A1). It may function either as P450 monooxygenase or as P420 farnesene-synthase. It has been shown that the farnesene-synthase activity predominates at pH 5.5–6.5 and in the presence of bivalent cations (Mg^2+^, Mn^2+^, Ca^2+^), while at pH 7.0–8.2, it functions as the monooxygenase, oxidizing epi-isozizaen first to albaflavenol, and then to albaflavenone (Moody et al., 2012). Conformation and final enzymatic activity of CYP170A1 is thus affected by the pH of the environment. We presume that *S. coelicolor* may exploit the dual pH-dependent activity of the enzyme in order to detect optimal external conditions. Therefore, we tested whether the biosynthetic activity is dependent on the pH of the medium during germination. For the experiment we performed the same R3 medium with a pH of either 7.2 or 6.0. Although we were not able to directly demonstrate the production of β-farnesene, we proved that the spectrum of detected substances significantly differed. Further experiments are required to confirm the expected pH-dependent signaling activity of the albaflavenon/β-farnesene systems.

### Germicidin A

Gcs protein (a polyketide synthase type III, PKS III) is involved in the germicidin biosynthesis in *S. coelicolor* (Chemler et al., 2012). However, expression of its gene sco7221 during germination was below the detection limit (Strakova et al., 2013). The germicidin biosynthesis could also be related to the activity of other genes sco7670-7671, whose expression is active in germination (Strakova et al., 2013).

Germicidin A production has previously been described in germination spores and in the stationary growth stage of *S. viridochromogenes* (Hirsch and Ensign, 1978; Petersen et al., 1993). It was also isolated after more than 24 h of submerged cultivation of *S. coelicolor* and other streptomyces (Petersen et al., 1993; Aoki et al., 2011; Ma et al., 2017). The results of our work, however, show for the first time that germicidin A is produced by germlings of *S. coelicolor* (in R3 medium with glycerol or mannitol, and in AM medium with glucose or glycerol). In contrast, germicidin B in germinating *S. coelicolor* was not produced, which is consistent with the results reported in *S. viridochromogenes* (Petersen et al., 1993). On the other hand, both polyketides, germicidin A and germicidin B, were detected here in samples from the stationary phase (in both, R3 medium with glycerol, or mannitol and NMMP medium with glucose or mannitol).

Germicidins belong to a richly represented group of α-pyrone natural substances found in bacteria, fungi, plants, and animals (Schaberle, 2016). Pyrones have many biological effects and signal molecules for *quorum sensing* can be found among them (Brachmann et al., 2013). Germicidin A is a known reversible inhibitor of spore germination; it prevents the germination at very low concentrations of 40 pg mL^−1^; i.e., only 2,400 molecules per spore (Petersen et al., 1993). We verified this biological effect in *S. coelicolor*, where germicidin A had a marked adverse effect on germination already at as low a concentration in medium as 4 μg mL^−1^. It is known that germicidin A affects the respiration of spores and mycelia by interacting with the membrane Ca^2+^-ATPase, inactivating the enzyme. By this mechanism, germicidin not only prevents spores from generating sufficient energy for germination but also inhibits hyphal growth (Eaton and Ensign, 1980; Grund and Ensign, 1985; Aoki et al., 2011). Germicidin A also exhibits antibacterial activity against gram-positive bacteria such as *Bacillus subtilis, Arthrobacter crystallopoietes*, or *Mycobacterium smegmatis* (Grund and Ensign, 1985; Aoki et al., 2011).

Because of its inhibitory effect, the production of germicidin during germination in optimal conditions might, at first glance, seem surprising. Its production by germinating spores might help to co-ordinate germination within the population. Its self-regulating function could maintain a portion of spores in their dormant state for a prolonged period as a reserve if the environment proves to be unfavorable or when germination occurs in higher spore densities. Conversely, the ungerminated spores can be further propagated in the environment and, after overcoming the reversible inhibition, can spread in new niches.

### Chalcone

The detection of chalcone in *Streptomyces* has not yet been proven to our knowledge. Its tentative identification presented here is based on the accurate mass of analyzed supernatants from the stationary phase and germination in R3 medium with glycerol or mannitol. Chalcones are intermediates of flavonoid biosynthesis where the key role in their synthesis plays 1,3,6,8-tetrahydroxynaphthalene-synthase. One of its precursors is the naringenin-chalcone, whose involvement in the flavonoid naringenin biosynthesis was described in *S. clavuligerus* (Alvarez-Alvarez et al., 2015). The enzyme, which belongs to PKS III, is closely related to the plant chalcone synthase (Izumikawa et al., 2003). Its sco1206 gene in *S. coelicolor* is expressed during germination (Strakova et al., 2013).

Chalcone is apparently an instrument of interspecies interaction, as there are referred its antifungal, phytotoxic and insecticidal effects are outlined (Diaz-Tielas et al., 2012). Chalcone could also function as a signaling molecule in a symbiotic relationship, such as 4,4′-dihydroxy-2′-methoxychalcone produced by legumes that induce transcription of *nod* genes in symbiotic rhizobacteria. The products of these genes, Nod factors, are involved in the symbiosis where rhizobacteria produce nitrogen for plants (Maxwell et al., 1989).

Interestingly, other flavonoids - quercetin, kaempferol, and myricetin—are known to stimulate pollen germination in *Nicotiana tabacum* L. (Ylstra et al., 1992). Therefore, we expected that chalcones produced by germinating spores would stimulate germination under favorable environmental conditions. Experimentally, however, we verified the opposite as the chalcone of *S. coelicolor* remarkably inhibited germination. At a concentration of 300 μg mL^−1^, it completely suppressed growth and at 8 μg mL^−1^ and lower the compound visibly inhibited spore germination, colony differentiation, and actinorhodin production on solid medium. Electron microscopic images taken from liquid cultivation revealed disrupted germ tubes. This finding correlates with a described activity of chalcone which was shown to interfere with cell membrane of *Staphylococcus aureus* (Sivakumar et al., 2009).

## Conclusion

In this work the production of secondary metabolites in germinating streptomycetes is systematically analyzed for the first time. Our investigation was based on the hypothesis that germinating spores exploit intercellular communications (*quorum sensing*) to support a coordinated development in its early stage as well as interspecies communication (*quorum quenching*) to suppress metabolic activities of competing microflora (Chen et al., 2000). This work succeeds the previous transcriptomic analysis of germination in streptomyces (Strakova et al., 2013), which has shown the expression of genes from different antibiotic clusters. Here using LC-MS, we detected three potentially important secondary metabolites—sesquiterpenoid albaflavenone, and polyketides germicidin A, and chalcone—that are synthesized during spore germination of *S. coelicolor*. All three detected compound possess capacities to suppress competitive microflora at the early stage of development. Their biosynthetic pathways are simple, having only a few reaction steps that do not require complex precursors.

Albaflavenone had been previously detected only in the stationary phase of growth of certain streptomycetes (Gurtler et al., 1994; Moody et al., 2012). It exhibits an antibacterial effect and could serve as a germination signal for coordinated development or as a factor of interspecific communication (these suggestions are not proven here). In contrary, the two other compounds revealed inhibitory effects on germination that may explain slower germination rate and less synchronicity in the model *S. coelicolor*, in comparison with other *Streptomyces* species, such as *S. viridochromogenes*. Our data are consistent with the known inhibitory effect of the supernatant of *S. coelicolor* on its own germination (Xu and Vetsigian, 2017).

The widespread autoregulator of germination, germicidin A, is known to be produced by germinating spores of *S. viridochromogenes*. Here it was shown that the germlings of *S. coelicolor* are also capable of its production. Chalcone is probably one of the precursors of biosynthesis of a not yet described flavonoid in *S. coelicolor*. During germination it functions as a germination inhibitor that may serve as a means of interspecies communication.

## Author contributions

MČ managed all experiments and evaluated data; MČ and KP performed analytical samples; MČ and ZK performed LC-MS measurements; KŠ and NB performed cultures and experimental design for germination; OB and OK performed the electronmicroscopy; JB concieved the project and wrote the manuscript.

### Conflict of interest statement

The authors declare that the research was conducted in the absence of any commercial or financial relationships that could be construed as a potential conflict of interest.
